# Targeting Rac1 Signaling Inhibits Streptococcal M1 Protein-Induced CXC Chemokine Formation, Neutrophil Infiltration and Lung Injury

**DOI:** 10.1371/journal.pone.0071080

**Published:** 2013-08-12

**Authors:** Songen Zhang, Milladur Rahman, Su Zhang, Lei Song, Heiko Herwald, Henrik Thorlacius

**Affiliations:** 1 Department of Clinical Sciences, Section for Surgery, Lund University, Malmö, Sweden; 2 Section for Clinical and Experimental Infection Medicine, Lund University, Lund, Sweden; University of Leuven, Rega Institute, Belgium

## Abstract

Infections with *Streptococcus pyogenes* exhibit a wide spectrum of infections ranging from mild pharyngitis to severe Streptococcal toxic shock syndrome (STSS). The M1 serotype of *Streptococcus pyogenes* is most commonly associated with STSS. In the present study, we hypothesized that Rac1 signaling might regulate M1 protein-induced lung injury. We studied the effect of a Rac1 inhibitor (NSC23766) on M1 protein-provoked pulmonary injury. Male C57BL/6 mice received NSC23766 prior to M1 protein challenge. Bronchoalveolar fluid and lung tissue were harvested for quantification of neutrophil recruitment, edema and CXC chemokine formation. Neutrophil expression of Mac-1 was quantified by use of flow cytometry. Quantitative RT-PCR was used to determine gene expression of CXC chemokines in alveolar macrophages. Treatment with NSC23766 decreased M1 protein-induced neutrophil infiltration, edema formation and tissue injury in the lung. M1 protein challenge markedly enhanced Mac-1 expression on neutrophils and CXC chemokine levels in the lung. Inhibition of Rac1 activity had no effect on M1 protein-induced expression of Mac-1 on neutrophils. However, Rac1 inhibition markedly decreased M1 protein-evoked formation of CXC chemokines in the lung. Moreover, NSC23766 completely inhibited M1 protein-provoked gene expression of CXC chemokines in alveolar macrophages. We conclude that these novel results suggest that Rac1 signaling is a significant regulator of neutrophil infiltration and CXC chemokine production in the lung. Thus, targeting Rac1 activity might be a potent strategy to attenuate streptococcal M1 protein-triggered acute lung damage.

## Introduction

Streptococcal toxic shock syndrome (STSS) is associated with a mortality rate surpassing 50% [1], [2]. The most feared complication in STSS is acute lung damage causing impaired gaseous exchange [3]. Due to a lack of understanding of the mechanisms behind lung injury in STSS, current treatment of patients suffering from STSS is largely limited to antibiotics and supportive care. *S. pyogenes* contains a wide spectrum of virulence factors, including M proteins. At present, more than 80 different M serotypes have been described in *S. pyogenes* [2], [4]. It is generally held that the M1 serotype is most frequently associated with STSS [1]. Interestingly, statins, which are mainly used to lower lipid levels in patients with cardiovascular diseases, have been reported to reduce mortality in patients with severe infections and sepsis although the mechanisms remain elusive [5], [6]. In fact a recent study showed that simvastatin protected against M1 protein-induced lung inflammation and tissue damage in mice [7]. Statins control synthesis of cholesterol via inhibition of HMG-CoA reductase, which is the rate-limiting enzyme in the formation of mevalonate. Mevalonate is not only a cholesterol precursor but also a precursor for isoprenoid pyrophosphates used for protein isoprenylation [6], [8]. Protein isoprenylation modifies small G-proteins, such as Rho A–C, Cdc42, and Rac1, which is important for their function by enabling localization at cell membranes [9], [10]. A previous study showed that the downstream effector of Rho proteins, Rho-kinase, plays a significant role in the regulation of M1 protein-induced lung damage [11]. Rac1 is a ubiquitously expressed signal transducer regulating numerous processes related to inflammatory reactions, such as cell adhesion, chemotaxis, vascular permeability and cytoskeletal reorganization [12]. For example, targeting Rac1 signaling has been demonstrated to exert anti-inflammatory effects in models of reperfusion injury, endotoxemia and acute pancreatitis [13], [14], [15]. However, the potential role of Rac1 in controlling pathological inflammation in M1 protein-induced lung damage is not known.

Accumulating data have convincingly demonstrated that M1 protein is a potent activator of innate immune cells, such as neutrophils and monocytes [2], [4]. Neutrophils constitute the first line of defense against invading microbes but overwhelming activation and accumulation of neutrophils is also considered to be a rate-limiting step in septic lung damage [16], [17]. Specific adhesion molecules, such as P-selectin and Mac-1, mediate interactions between circulating neutrophils and endothelial cells in the lung [18], [19]. Numerous studies have shown that M1 protein effectively up-regulates Mac-1 expression on neutrophils. CXC chemokines, including CXCL1 and CXCL2, control localization of neutrophils at sites of tissue injury [20]. A recent study demonstrated that M1 protein-provoked pulmonary accumulation of neutrophils is dependent on the generation and action of CXC chemokines [7]. Thus, the adhesive and chemokine-mediated mechanisms behind accumulation of neutrophils in the lung are relatively well known, but the signaling pathways regulating M1 protein-induced neutrophil infiltration and lung damage remain elusive.

Based on the considerations above, the aim of the present investigation was to define the functional significance of Rac1 in controlling CXC chemokine production, neutrophil Mac-1 expression and recruitment as well as lung damage provoked by streptococcal M1 protein.

## Materials and Methods

### Animals

Male C57BL/6 mice weighing 20 to 25 g were used for experiments and kept under standard laboratory conditions, maintained on a 12–12 hour light dark cycle and fed a laboratory diet and water ad libitum. Animals were anesthetized with 75 mg of ketamine hydrochloride (Hoffman-La Roche, Basel, Switzerland) and 25 mg of xylazine (Janssen Pharmaceutica, Beerse, Belgium) per kg body weight. All experimental procedures were performed in accordance with the legislation on the protection of animals and were approved by the Regional Ethical Committee for Animal Experimentation at Lund University, Sweden.

### Experimental Model

M1 protein was purified from the isogenic mutant MC25 strain (derived from the AP1 *Streptococcus pyogenes* strain 40/58 from the WHO Collaborating Centre for references and Research on Streptococci, Institute of Hygiene and Epidemiology, Prague, Czech Republic) as described previously [21]. Mice were intravenously injected with 15 µg of M1 protein in phosphate-buffered saline (PBS). M1 protein was purified from a mutated *Streptococcus pyogenes* strain making the likelihood of endotoxin contamination close to zero. Sham mice received PBS intravenously (i.v.) only. Treatment with the Rac1 inhibitor, NSC23766 (0.5 or 5 mg/kg, Tocris Bioscience, Bristol, UK) or vehicle (PBS) were administered intraperitoneally (i.p.) 10 min prior to M1 protein challenge. NSC23766 was dissolved in sterile distilled water and diluted in PBS just before injection. Animals were re-anesthetized 4 h after M1 protein challenge. The left lung was ligated and excised for edema measurement. The right lung was used for collecting bronchoalveolar lavage fluid (BALF) to quantify neutrophils. Then, the lung was excised and one lobe was fixed in formaldehyde for histology and the remaining lung tissue was snap-frozen in liquid nitrogen and stored at −80°C for later myeloperoxidase (MPO) assays and enzyme-linked immunosorbent assay (ELISA) as described subsequently.

### Rac1 Activity

Rac1 activation assay was performed by using Active Rac1 Pull-Down and Detection Kit (Thermo Scientific, USA). 50 mg of lung tissue were homogenatedin lysis buffer, the samples were centrifuged at 16 000 g at 4°C for 15 minutes. 10 µl of each supernatant were measured protein content by using Pierce BCA Protein Assay Reagent (Pierce Biotechnology, Rockford, IL, USA) and the rest of the volume was analyzed by pull-down assay. Add 100 µl of the 50% Glutathione Resin mixed with the agarose beads. Each sample was incubated with 20 µg of GST-human Pak1-PBD at 4°C for 1 hour. Next, the samples were separated by electrophoresis, transferred onto nitrocellulose membrane, and probed with anti-Rac1 antibody. After washing 5 times in TBST, the membranes was incubated with the anti-mouse IgG-HRP Conjugate solution (1∶20000, Pierce Goat Anti-Mouse IgG), exposed in the CCD camera and the resultant signal was quantified by using densitometer (ChemiDoc XRS System, Bio-Rad, USA).

### Systemic Leukocyte Count

Blood was collected from the tail vein and mixed with Turks solution (0.2 mg gentian violet in 1 ml glacial acetic acid, 6.25% v/v) in a 1∶20 dilution. Leukocytes were identified as monomorphonuclear (MNLs) and polymorphonuclear (PMNLs) cells in a Burker chamber.

### Lung Edema

The left lung was excised, washed in PBS, gently dried using a blotting paper and weighed. The tissue was then dried at 60°C for 72 h and re-weighed. The change in the ratio of wet weight to dry weight was used as indicator of lung edema formation.

### MPO Activity

Lung tissue was thawed and homogenized in 1 ml of 0.5% hexadecyltrimethylammonium bromide. Samples were freeze-thawed, after which the MPO activity of the supernatant was determined spectrophotometrically as the MPO-catalysed change in absorbance in the redox reaction of H_2_O_2_ (450 nm, with a reference filter 540 nm, 25°C) as previously described [18]. Values were expressed as MPO units per g tissue.

### BALF

Animals were placed supine and the trachea was exposed by dissection. A catheter was inserted into the trachea. BALF was collected by 5 washes of 1 ml of PBS containing 5 mM EDTA. The numbers of MNL and PMNL cells were counted in a Burker chamber.

### ELISA

Levels of CXCL1 and CXCL2 in lung homogenates were analyzed by using double antibody Quantikine ELISA kits (R & D Systems, Europe, Abingdon, Oxon, UK) using recombinant murine CXCL1 and CXCL2 as standards. The lower limit of the assay was 0.5 pg/ml.

### Flow Cytometry

For analysis of surface molecules expression on circulating neutrophils, blood was collected (1∶10 acid citrate dextrose) 4 h after M1 protein challenge and incubated (10 min, RT) with an anti-CD16/CD32 antibody blocking Fcγ III/II receptors to reduce non-specific labelling and then incubated with PE-conjugated anti-Gr-1 (clone RB6-8C5, rat IgG2b, eBioscience, San Diego, CA, USA), and FITC-conjugated anti-Mac-1 (clone M1/70, integrin α_M_ china, rat IgG2b). The mean fluorescence intensity (MFI) was determined by comparisons to an isotype control antibody (FITC-conjugated rat IgG2b). All antibodies were purchased from BD Biosciences Pharmingen, San Jose, CA, USA except indicated. Cells were fixed and erythrocytes were lysed by BD lysis buffer (BD Biosciences, USA) and then neutrophils were recovered following centrifugation. Flow-cytometric analysis was performed by first gating the neutrophil population of cells based on forward and side scatter characteristics and then Mac-1 expression was determined on Gr-1^+^ cells in this gate on a FACS Calibur flow cytometer (Becton Dickinson, Mountain View, CA, USA). A viable gate was used to exclude dead and fragmented cells.

### Histology

Lung samples were fixed in 4% formaldehyde phosphate buffer overnight and then dehydrated and paraffin-embedded. Six µm sections were stained with haematoxylin and eosin. Lung injury was quantified in a blinded manner by adoption of a pre-existing scoring system as described [22], including size of alveolar collapse, thickness of alveolar septum, alveolar fibrin deposition and neutrophil infiltration graded on a 0 (absent) to 4 (extensive) scale. In each tissue sample, 5 random areas were scored and mean value was calculated. The histology score is the sum of all 4 parameters.

### Quantitative RT-PCR

Alveolar macrophages were isolated as previously described [23]. Animals were challenged with M1 protein from 30 min and then was total RNA isolated from alveolar macrophages by use of RNeasy Mini Kit (Qiagen, West Sussex, UK) and treated with RNase-free DNase (DNase I; Amersham Pharmacia Biotech, Sollentuna, Sweden) to remove potential genomic DNA contaminants. RNA concentrations were determined by measuring the absorbance at 260 nm. Each cDNA was synthesized by reverse transcription from 10 µg of total RNA by use of StrataScript First-Strand Synthesis System and random hexamers primers (Stratagene, AH diagnostics, Stockholm, Sweden). Real-time PCR was performed using a Brilliant SYBRgreen QPCR master mix and MX 3000P detection system (Stratagene). The primers sequences of CXCL1, CXCL2 and β-actin were as follows: CXCL1 (f) 5′-GCT TCC TCG GGC ACT CCA GAC -3′, CXCL1 (r) 5′-TTA GCC TTG CCT TTG TTC AGT AT -3′; CXCL2 (f) 5′-GCC AAT GAG CTG CGC TGT CAA TGC -3′, CXCL2(r) 5′-CTT GGG GAC ACC TTT TAG CAT CTT -3′; β-actin (f) 5′-ATG TTT GAG ACC TTC AAC ACC-3′, β-actin (r) 5′-TCT CCA GGG AGG AAG AGG AT-3′. Standard PCR curves were generated for each PCR product to establish linearity of the RT-PCR reaction. PCR amplifications were performed in a total volume of 50 µl, containing 25 µl of SYBRgreen PCR 2 × master mix, 2 µl of 0.15 µM each primer, 0.75 µl of reference dye, and one 1 µl cDNA as a template adjusted up to 50 µl with water. PCR reactions were started with 10 min denaturing temperature of 95°C, followed by a total of 40 cycles (95°C for 30 s and 55°C for 1 min) and 1 min of elongation at 72°C. The relative differences in expression between groups were expressed by using cycling time values. Cycling time values for the specific target genes were first normalized with that of β-actin in the same sample, and then relative differences between groups were expressed as percentage of control.

### Cell Lines

The murine macrophage line RAW 264.7 was cultured in Dulbecco’s modified Eagle’s medium (DMEM; Gibco BRL, San Fransisco, CA, USA) containing 10% heat-inactivated fetal bovine serum (FBS; Gibco BRL) supplemented with penicillin (100 U/ml; Gibco BRL) and streptomycin (100 µg/ml; Gibco BRL). The polyoma transformed murine endothelioma cell line eEnd2 was cultured in DMEM supplemented with FBS, penicillin and streptomycin as above. The eEnd2 cell line was a kind gift of Dr. Dietmar Vestweber, Max Planck Institute for Molecular Biomedicine, Münster, Germany. 2×10^5^ RAW264.7 and eEND2 cells per well were incubated for 1 h with 10 µM of NSC23766 prior to challenge with 0.5 µg/ml of M1 protein for 4 h. The concentrations of CXCL2 in the cell culture supernatants were analyzed by use of ELISA as described above.

### Statistics

Data are presented as mean values ± standard errors of the means (SEM). Statistical evaluations were performed using Kruskal-Wallis one-way analysis of variance on ranks followed by multiple comparisons versus control group (Dunnett’s method). Mann Whitney rank-sum test was used for comparing two groups. *P*<0.05 was considered significant and *n* represents the number of animals.

## Results

### Rac1 Activity in the Lung

**Figure 1:**
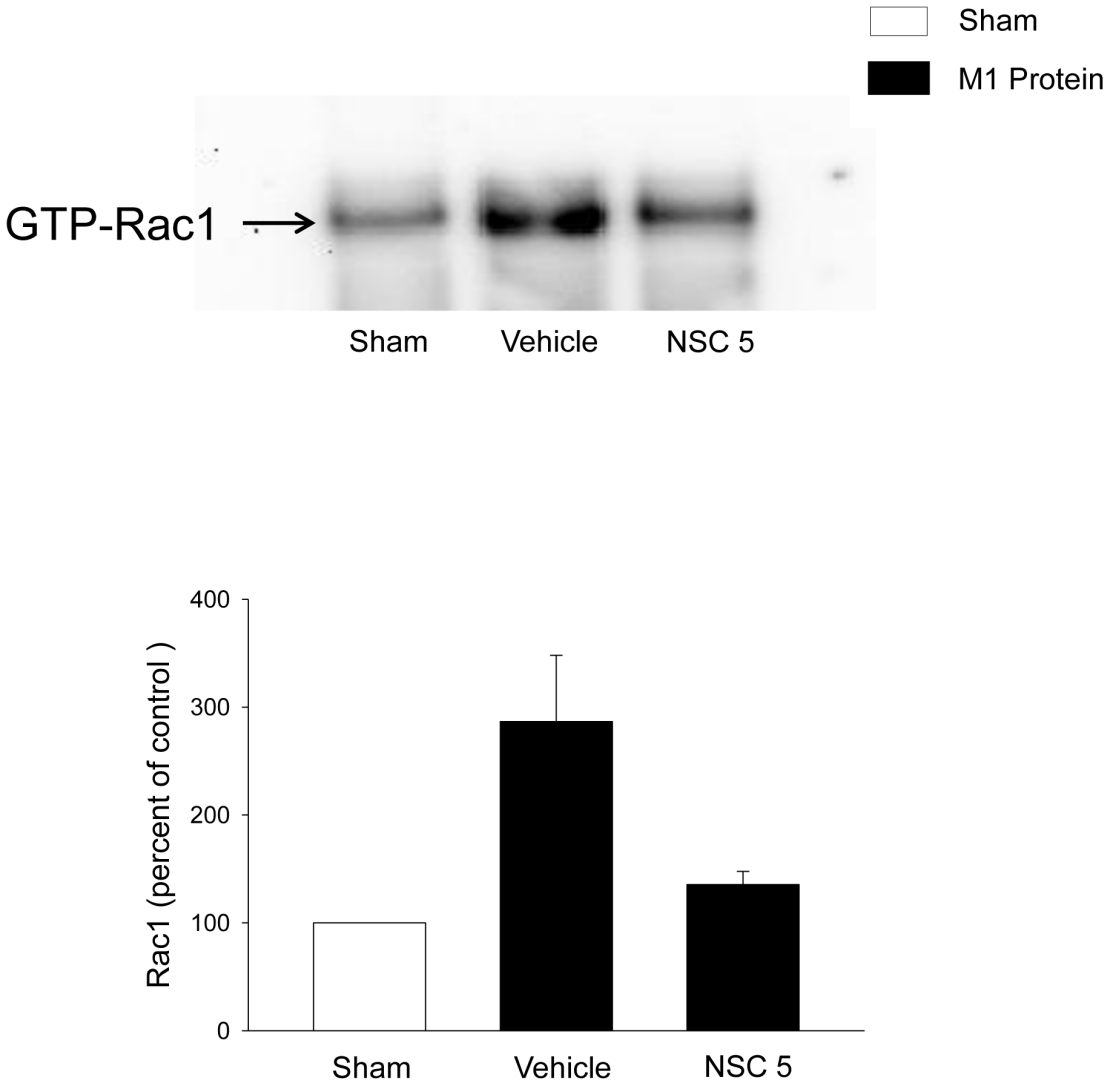
M1 protein-induced Rac1 activity in the lung. Mice were treated with the Rac1 inhibitor NSC23766 (5 mg/kg) or vehicle (PBS) 10 min prior to M1 protein injection. Mice treated with PBS served as sham animals. Samples were harvested 4 h after M1 protein challenge. *n* = 3.

In order to study the activation of Rac1 in the lung after challenge with M1 protein and the effect of NSC23766, lungs from sham and M1 protein-treated mice were harvested for quantification of Rac1 activation. M1 protein enhanced the active form (GTP binding form) of Rac1 (Fig. 1). Administration of NSC23766 reduced M1 protein-provoked activation of Rac1 (Fig. 1).

### Lung Edema and Injury

**Figure 2:**
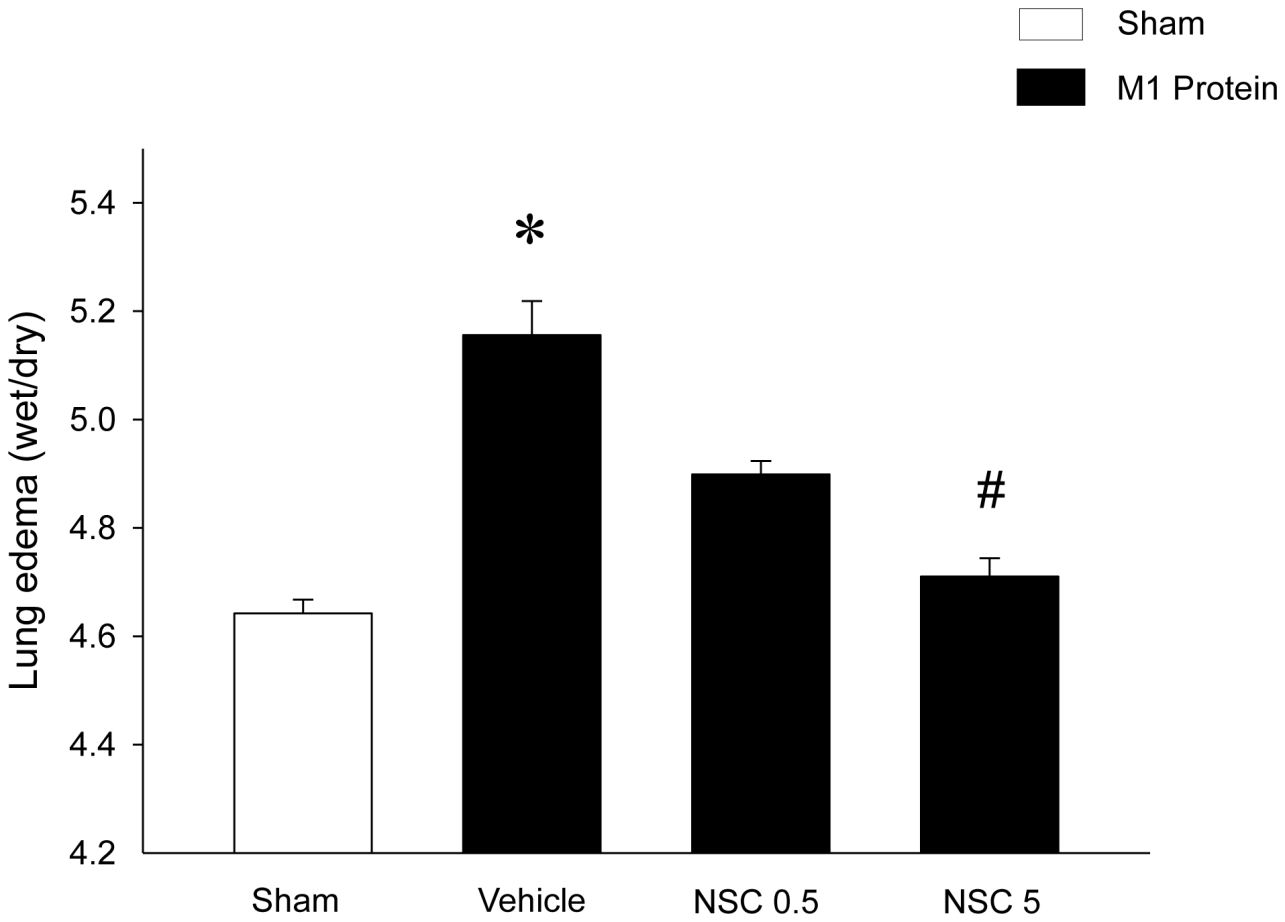
Rac1 regulates M1 protein-induced edema formation in the lung. Mice were treated with the Rac1 inhibitor NSC23766 (0.5 or 5 mg/kg) or vehicle (PBS) 10 min prior to M1 protein injection. Mice treated with PBS served as sham animals. Samples were harvested 4 h after M1 protein challenge. Data represents mean ± SEM, *P < 0.05 *vs.* Sham and **^#^** *P* < 0.05 *vs.* Vehicle+M1 protein, *n = *5.

**Figure 3:**
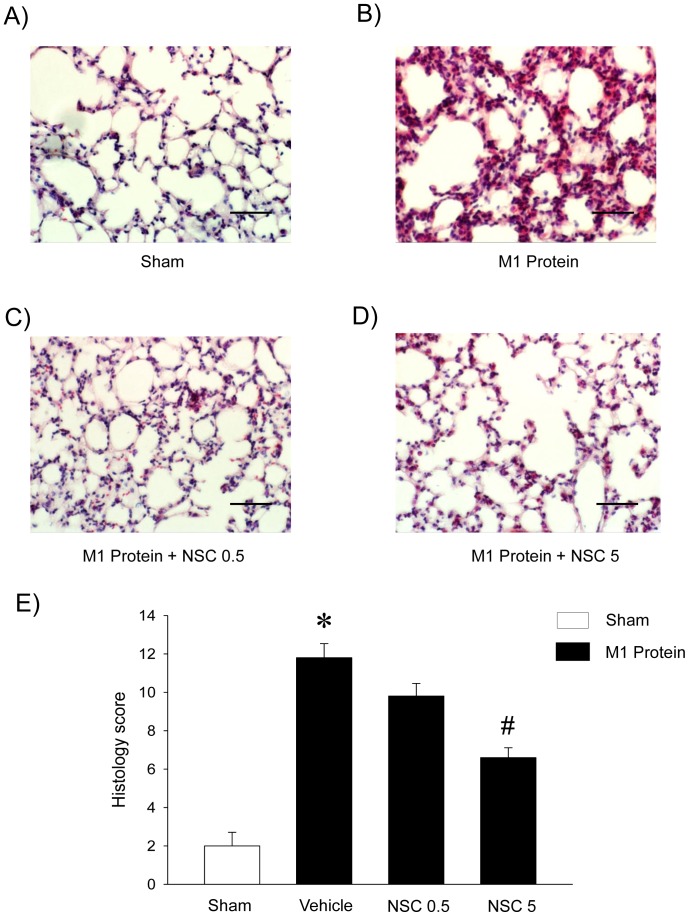
Rac1 regulates M1 protein-induced lung damage. Representative haematoxylin & eosin sections of lung. Sham animals were treated with PBS only. Separate mice were pretreated with vehicle (PBS) and 0.5 or 5 mg/kg of the Rac1 inhibitor NSC23766 10 min prior to M1 protein administration. Samples were harvested 4 h after M1 protein challenge. Scale bar indicates 100 µm. Histology score of lung injury. Data represents mean ± SEM, *P < 0.05 *vs.* Sham and **^#^** *P* < 0.05 *vs.* Vehicle+M1 protein, *n = *5.

M1 protein challenge induced significant lung damage characterized by increased lung edema formation (Fig. 2). Thus, lung wet:dry ratio increased from 4.6±0.03 in sham mice up to 5.2±0.06 in M1 protein-treated mice (Fig. 2). Administration of the Rac1 inhibitor NSC23766 (5 mg/kg) decreased lung wet:dry ratio down to 4.7±0.03 in animals challenged with M1 protein (Fig. 2). Thus, inhibition of Rac1 signaling attenuated M1 protein-induced lung edema by 87%. In addition, morphologic examination revealed normal lung architecture in sham-operated mice (Fig. 3A), whereas M1 protein caused destruction of the lung tissue structure characterized by interstitial edema, capillary congestion and neutrophil accumulation (Fig. **3**B). We observed that targeting Rac1 activity decreased M1 protein-induced changes of the microarchitecture and neutrophil accumulation in the lung (Fig. **3**D). Quantification of the morphological changes revealed that M1 protein enhanced the lung damage score and that administration of NSC23766 significantly reduced the M1 protein-evoked lung injury score (Fig. **3**E).

### Pulmonary Accumulation of Neutrophils

**Figure 4:**
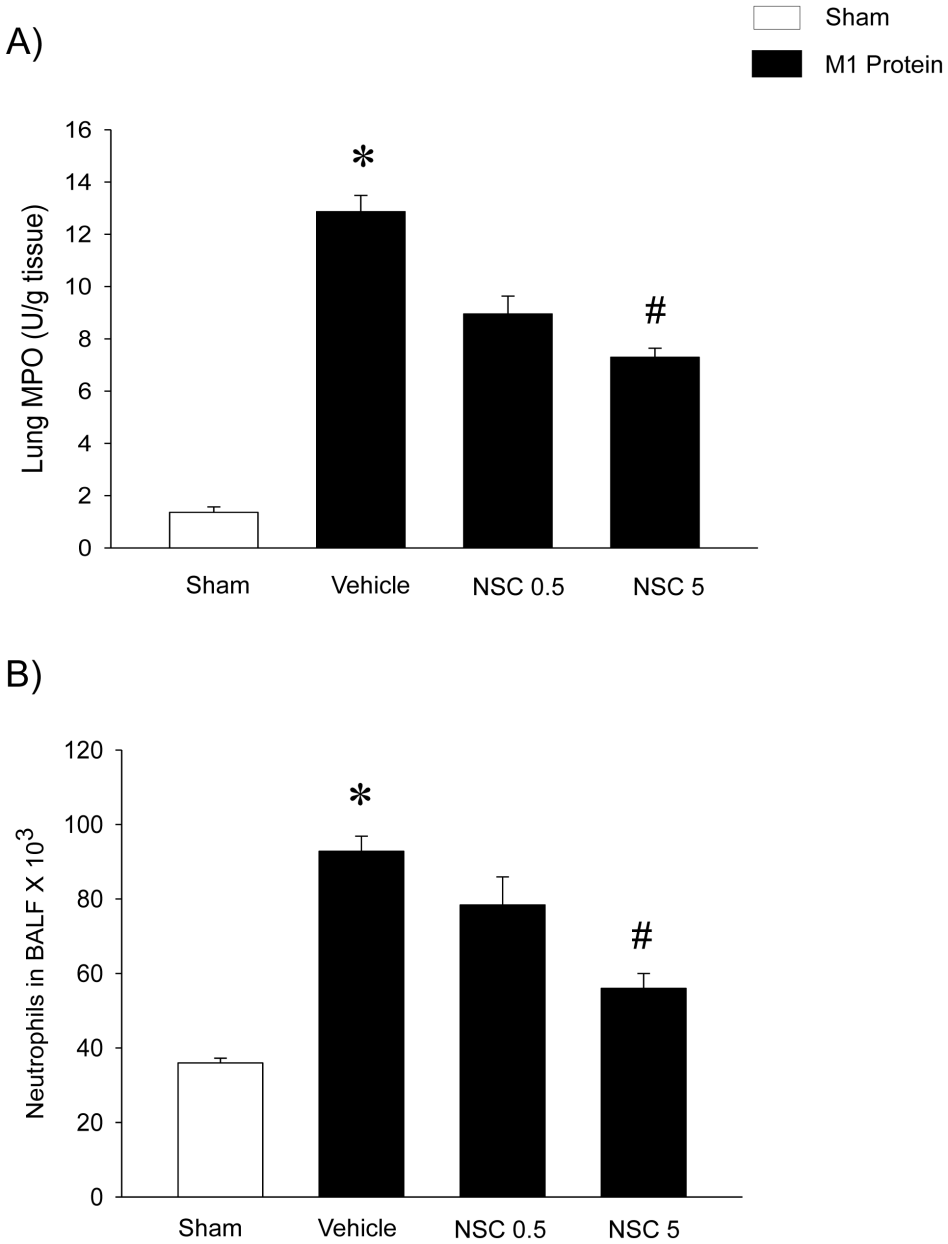
Rac1 regulates M1 protein-induced neutrophil infiltration in the lung. MPO levels and number of BALF neutrophils in the lung. Animals were treated with the Rac1 inhibitor NSC23766 (0.5 or 5 mg/kg) or vehicle (PBS) 10 min prior to M1 protein injection. Samples were harvested 4 h after M1 protein challenge. Mice treated with PBS served as sham animals. Data represents mean ± SEM, *P < 0.05 *vs.* Sham and **^#^** *P* < 0.05 *vs.* Vehicle+M1 protein, *n = *5.

Challenge with M1 protein enhanced lung activity of MPO by more than 9-fold (Fig. 4A). Rac1 inhibition decreased the M1 protein-induced increase in pulmonary levels of MPO by 48% (Fig. 4A). The number of BALF neutrophils was markedly increased 4 h after administration of M1 protein (Fig. 4B). We observed that treatment with NSC23766 decreased the number of pulmonary neutrophils from 92.8±4.1×10^3^ to 56.0±4.0×10^3^ in the lung, corresponding to a 65% reduction, 4 h after M1 protein challenge (Fig. 4B). In addition, we found that challenge with M1 protein decreased the number of circulating PMNLs and MNLs (Table 1). Rac1 inhibition significantly decreased this M1 protein-induced leukocopenia (Table 1).

**Table 1:** Systemic leukocyte differential counts.

	MNL	PMNL	Total
Sham	4.0±0.2	1.9±0.0	6.0±0.2
Vehicle+M1 Protein	1.1±0.1*	0.4±0.1*	1.5±0.2*
NSC23766 (0.5 mg/kg)+M1 Protein	2.5±0.3	1.1±0.2	3.6±0.3**^#^**
NSC23766 (5 mg/kg)+M1 Protein	3.8±0.4**^#^**	1.6±0.1**^#^**	5.4±0.5**^#^**

Blood was collected from sham animals receiving PBS intravenously only as well as mice were pretreated i.p. with vehicle (PBS) or NSC23766 10 min prior to M1 protein challenge for 4 h. Cells were identified as monomorphonuclear leukocytes (MNL) and polymorphonuclear leukocytes (PMNL). Data represents mean ± SEM, 10^6^ cells/ml and *n = *5.

* *P* < 0.05 *vs.* Sham and ^#^ *P* < 0.05 *vs.* Vehicle+M1 protein, *n = *5.

### Mac-1 Expression and CXC Chemokine Generation

**Figure 5:**
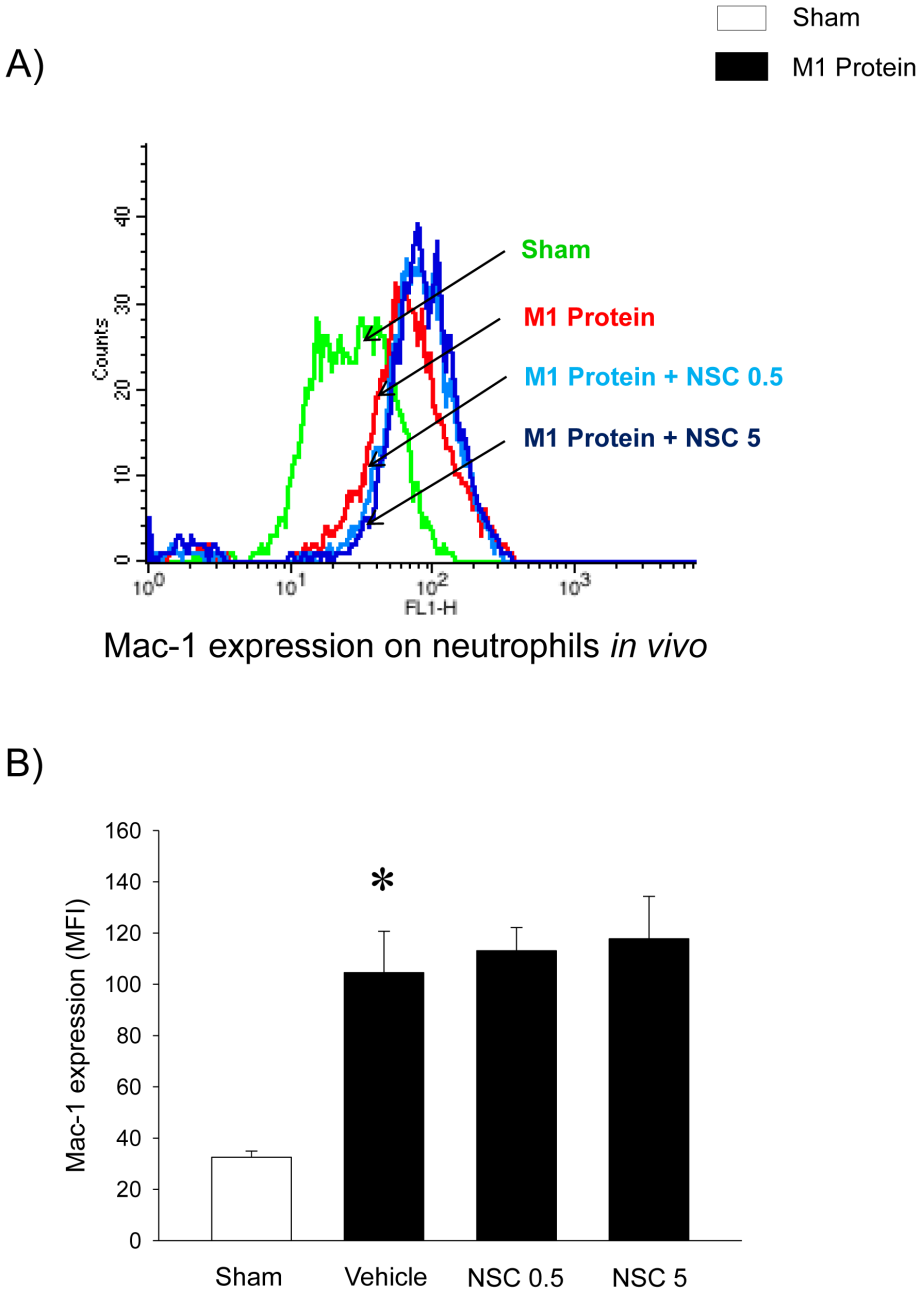
M1 protein-induced Mac-1 expression on neutrophils *in vivo*. Mac-1 expression on neutrophils in vehicle (PBS) or NSC23766 (0.5 or 5 mg/kg), treated animals 4 h after M1 protein injection. Fluorescence intensity is shown on the x-axis and cell counts on the y-axis. Data represents mean ± SEM, ^*^ *P* < 0.05 *vs.* Sham and ^#^ *P* < 0.05 *vs.* Vehicle+M1 protein, *n = *5.

**Figure 6:**
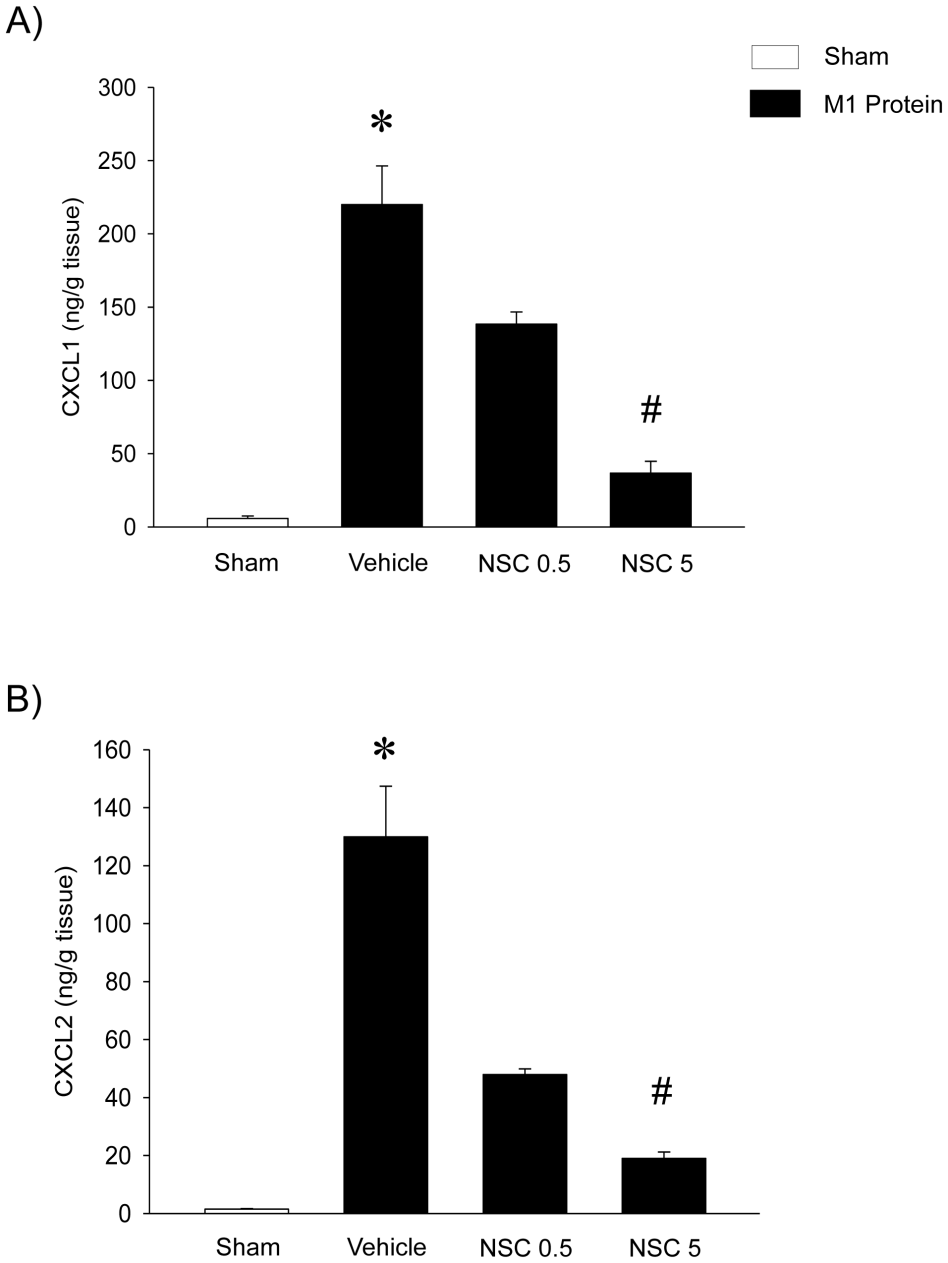
Rac1 regulates M1 protein-induced CXC chemokine formation in the lung. Animals were treated with the Rac1 inhibitor NSC23766 (0.5 or 5 mg/kg) or vehicle (PBS) 10 min prior to M1 protein injection. Mice treated with PBS served as sham animals. ELISA was used to quantify the levels of CXCL1 and CXCL2 in the lung of mice 4 h after M1 protein challenge. Data represents mean ± SEM, *P < 0.05 *vs.* Sham and **^#^** *P* < 0.05 *vs.* Vehicle+M1 protein, *n = *5.

**Figure 7:**
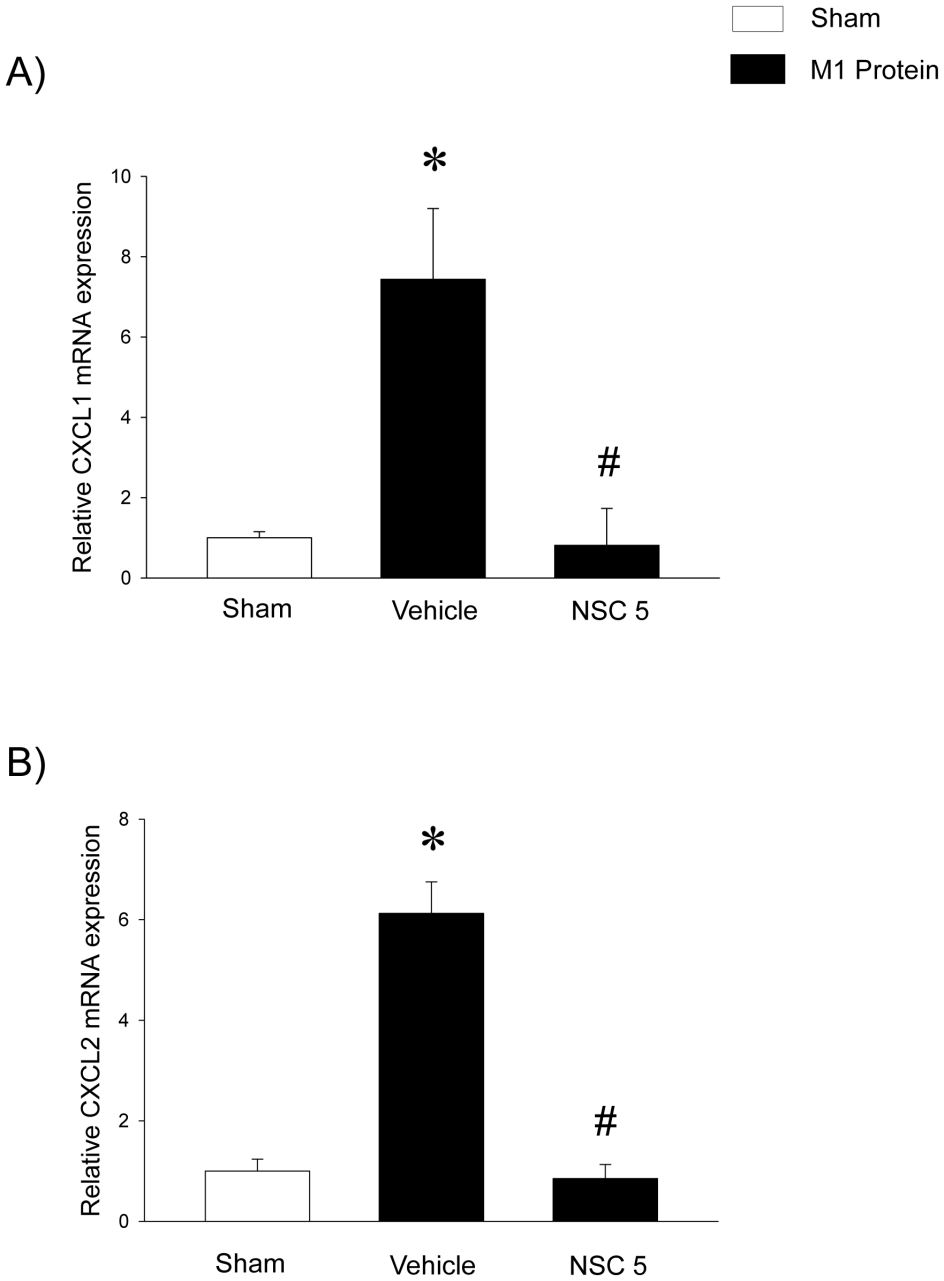
Rac1 regulates M1 protein-induced gene expression of CXC chemokines in alveolar macrophages. CXCL1 and CXCL2 in alveolar macrophages 30 min after M1 protein injection. Levels of CXCL1 and CXCL2 mRNA were normalized to mRNA levels of β-actin. Data represents mean ± SEM, *P < 0.05 *vs.* Sham and **^#^** *P* < 0.05 *vs.* Vehicle+M1 protein, *n = *5.

**Figure 8:**
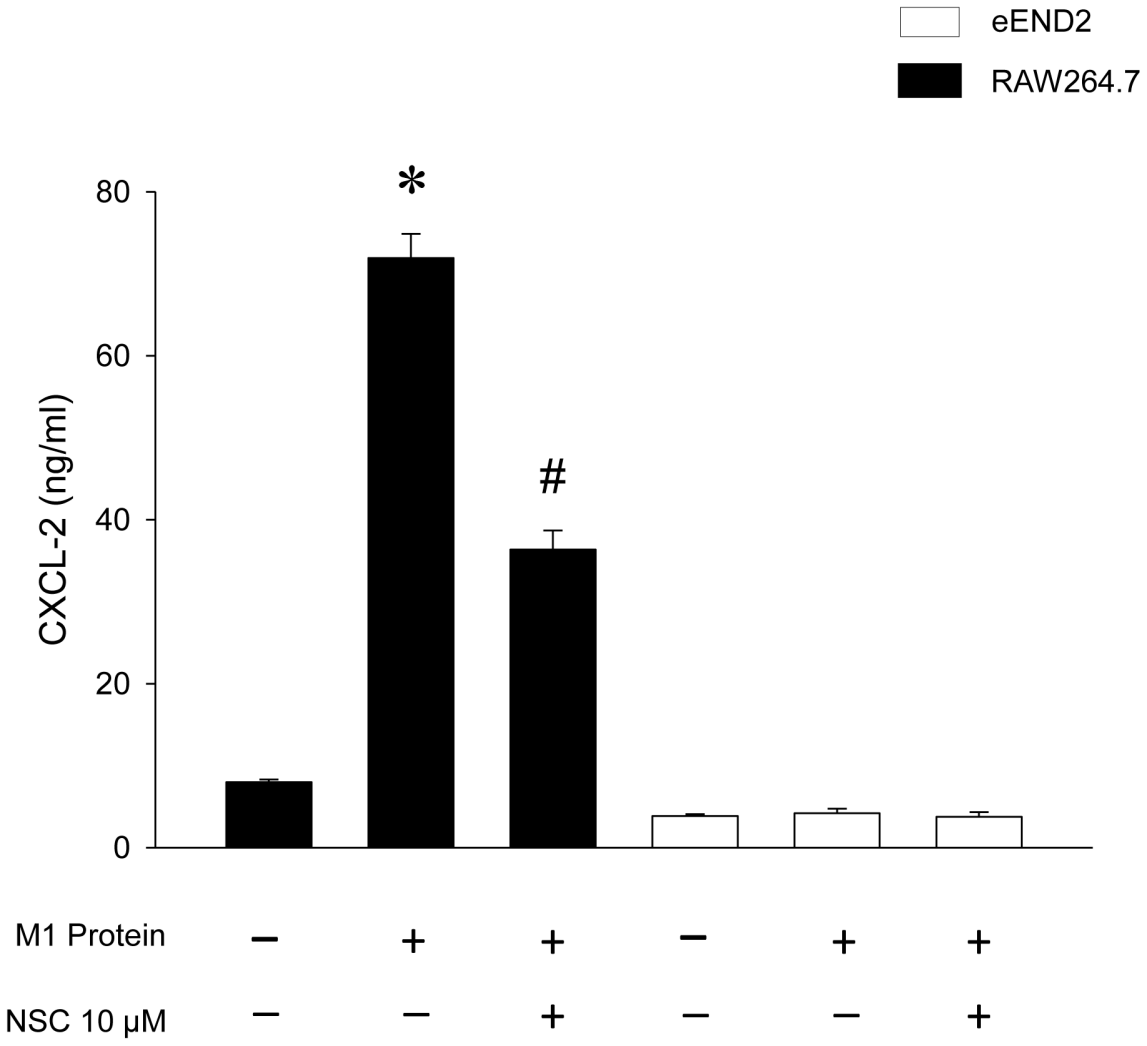
Macrophages (RAW264.7) and endothelial cells (eEND2) were co-incubated with 10 µM Rac-1 inhibitor NSC23766 1 h prior to M1 protein (0.5 µg/ml) stimulation. ELISA was used to quantify the levels of CXCL2 in the supernatants 4 h after M1 protein challenge. Data represents mean ± SEM, *P < 0.05 *vs.* Sham and ^#^ *P* < 0.05 *vs.* Vehicle+M1 protein, *n = *5.

Challenge with M1 protein markedly enhanced expression of Mac-1 on neutrophils compared to PBS-treated control mice (Fig. 5). It was found that inhibition of Rac1 activity had no effect on M1 protein-evoked increases of Mac-1 expression on the neutrophils (Fig. 5). Numerous studies have shown that CXC chemokines are potent regulators of neutrophil recruitment in the lung. Thus, we next analyzed the role of Rac1 signaling in controlling pulmonary generation of CXCL1 and CXCL2. Pulmonary levels of CXC chemokines were low but detectable in sham-operated animals whereas administration of M1 protein provoked a 38-fold and 88-fold increase in the lung levels of CXCL1 and CXCL2, respectively (Fig. 6). It was found that NSC23766 significantly decreased M1 protein-induced formation CXCL1 and CXCL2 by more than 85% (Fig. 6). We next isolated alveolar macrophages from the BALF in animals challenged with M1 protein and/or NSC23766. We observed that Rac1 inhibition greatly decreased M1 protein-induced mRNA levels of CXCL1 and CXCL2 in alveolar macrophages (Fig. 7). In separate experiments, we studied M1 protein-induced formation of CXCL2 in macrophages and endothelial cells. It was observed that M1 protein triggered clear-cut CXCL2 production in macrophages but not in endothelial cells (Fig. 8). Moreover, co-incubation of macrophages with NSC23766 significantly decreased M1 protein-evoked CXCL2 formation (Fig. 8).

## Discussion

The management of patients with STSS is a major challenge for clinicians, which is related to an incomplete understanding of the mechanisms behind in streptococcal-induced acute lung injury. Herein, we present findings showing a significant role of Rac1 signaling in lung injury caused by streptococcal M1 protein. Thus, inhibition of Rac1 activity decreased M1 protein-induced pulmonary infiltration of neutrophils and tissue damage. Moreover, these results indicate that Rac1 specification regulates M1 protein-evoked formation of CXC chemokines in lung macrophages in sepsis.

The M1 serotype of *Streptococcus pyogenes* is commonly associated with STSS and high mortality. During infection, *Streptococcus pyogenes* shed M1 protein from their surface, which subsequently cause widespread activation of the innate immune system. In the present study, we demonstrate for the first time that targeting Rac1 activity reduces edema formation and structural injury in the lungs of mice exposed to M1 protein, indicating that Rac1 plays a key role in controlling acute lung injury in streptococcal infections. This notion is also in line with recent findings demonstrating that inhibition of farnesyltransferase, an upstream regulator of Rac1 isoprenylation, reduces lung injury induced by M1 protein challenge [24]. Although this study provides the first direct data indicating a role of Rac1 in streptococcal lung injury, Yao et al. [14] reported that NSC23766 attenuates endotoxemic lung damage, supporting the concept that Rac1 exert pro-inflammatory effects in the lung. Knowing that simvastatin reduces streptococcal M1 protein-provoked lung damage and that statins prevent isoprenylation of small GTPases [7], our present results might help explain the lung protective effects of simvastatin in streptococcal infections.

Given that neutrophil infiltration is a key feature in M1 protein-provoked lung injury [20], it was of interest to investigate the impact of NSC23766 on pulmonary neutrophilia. In the present study, it was observed that NSC23766 reduced M1 protein-induced pulmonary MPO activity by 48%, suggesting that Rac1 activity plays a significant role in neutrophil infiltration in streptococcal infections. This Rac-1-dependent reduction in MPO activity correlated well with the decrease in the number of neutrophils in the alveolar space (65%) in M1 protein-treated mice. Considering the relationship between neutrophil accumulation and lung injury [16], it might be suggested that the lung protective impact of NSC23766 is due to the attenuation of neutrophil recruitment into the lung.

Tissue localization of neutrophils at sites of inflammation is a multistep process facilitated by specific adhesion molecules expressed on neutrophils, such as P-selectin glycoprotein ligand-1 (PSGL-1) and Mac-1. Consequently, we next asked whether Rac1 might control neutrophil activation and expression of Mac-1. However, it was found that Rac1 inhibition had no impact of Mac-1 up-regulation on neutrophils, indicating that the NSC23766-mediated reduction of neutrophil infiltration is not related to changes in neutrophil expression of Mac-1 in streptococcal infections. Orchestration of neutrophil trafficking in the extravascular space is coordinated by secreted CXC chemokines, including CXCL1 and CXCL2, which are the murine homologues of human interleukin-8 [25]. A functional role of CXC chemokines has been proposed in streptococcal infections [11], [26] and we have demonstrated that M1 protein is a potent stimulator of CXCL1 and CXCL2 production in the lung [20]. In the present study, we found that administration of NSC23766 greatly decreased CXCL1 and CXCL2 generation in the lung, suggesting that Rac1 signaling is an important regulator of CXC chemokine formation in the lung in response to M1 protein challenge. Additionally, it was observed that M1 protein markedly increased CXCL1 and CXCL2 mRNA levels in alveolar macrophages. Notably, administration of NSC23766 completely inhibited M1 protein-induced gene expression of CXCL1 and CXCL2 in alveolar macrophages, indicating that Rac1 activity mediates macrophage production of CXC chemokines in streptococcal infections. However, the decreased alveolar infiltration of neutrophils in NSC23766-treated animals could potentially also explain the reduced formation of CXC chemokines in lung macrophages in M1 protein-induced inflammation. We therefore examined the direct role of Rac1 in M1 protein-induced production of CXCL2 in macrophages. It was found that M1 protein was a potent inducer of CXCL2 production in macrophages and that NSC23766 markedly attenuated M1 protein-evoked CXCL2 formation. Although RAW 264.7 macrophages are not identical to alveolar macrophages, these findings further support the concept that a Rac1-CXC chemokine axis in macrophages is an important feature in M1 protein-induced lung injury.

In conclusion, our novel findings suggest that Rac1 signaling is a key component in the pathophysiology of acute lung injury triggered by streptococcal M1 protein. Inhibition of Rac1 activity reduces M1 protein-provoked neutrophil infiltration in the lung via inhibition of CXC chemokine formation in alveolar macrophages. These results suggest that targeting Rac1 signaling might be a useful approach in order to protect respiratory function in streptococcal infections.

## References

[1] Cunningham MW (2000) Pathogenesis of group A streptococcal infections. Clin Microbiol Rev 13: 470–511. 10885988 10.1128/cmr.13.3.470-511.2000 PMC88944

[2] Herwald H, Cramer H, Morgelin M, Russell W, Sollenberg U, et al (2004) M protein, a classical bacterial virulence determinant, forms complexes with fibrinogen that induce vascular leakage. Cell 116: 367–379. 15016372 10.1016/s0092-8674(04)00057-1

[3] Stevens DL (2003) Group A Streptococcal Sepsis. Curr Infect Dis Rep 5: 379–386. 13678567 10.1007/s11908-003-0017-7 PMC7101722

[4] Pahlman LI, Morgelin M, Eckert J, Johansson L, Russell W, et al (2006) Streptococcal M protein: a multipotent and powerful inducer of inflammation. J Immunol 177: 1221–1228. 16818781 10.4049/jimmunol.177.2.1221

[5] Beltowski J, Wojcicka G, Jamroz-Wisniewska A (2009) Adverse effects of statins - mechanisms and consequences. Curr Drug Saf 4: 209–228. 19534648 10.2174/157488609789006949

[6] Stancu C, Sima A (2001) Statins: mechanism of action and effects. J Cell Mol Med 5: 378–387. 12067471 10.1111/j.1582-4934.2001.tb00172.x PMC6740083

[7] Zhang S, Rahman M, Qi Z, Herwald H, Thorlacius H (2011) Simvastatin regulates CXC chemokine formation in streptococcal M1 protein-induced neutrophil infiltration in the lung. Am J Physiol Lung Cell Mol Physiol 300: L930–939. 21441352 10.1152/ajplung.00422.2010

[8] Bonetti PO, Lerman LO, Napoli C, Lerman A (2003) Statin effects beyond lipid lowering–are they clinically relevant? Eur Heart J 24: 225–248. 12590901 10.1016/s0195-668x(02)00419-0

[9] Van Aelst L, D'Souza-Schorey C (1997) Rho GTPases and signaling networks. Genes Dev 11: 2295–2322. 9308960 10.1101/gad.11.18.2295

[10] Brown JH, Del Re DP, Sussman MA (2006) The Rac and Rho hall of fame: a decade of hypertrophic signaling hits. Circ Res 98: 730–742. 16574914 10.1161/01.RES.0000216039.75913.9e

[11] Zhang S, Rahman M, Herwald H, Qi Z, Jeppsson B, et al (2012) Streptococcal M1 protein-provoked CXC chemokine formation, neutrophil recruitment and lung damage are regulated by Rho-kinase signaling. J Innate Immun 4: 399–408. 22433673 10.1159/000336182 PMC6741568

[12] Etienne-Manneville S, Hall A (2002) Rho GTPases in cell biology. Nature 420: 629–635. 12478284 10.1038/nature01148

[13] Harada N, Iimuro Y, Nitta T, Yoshida M, Uchinami H, et al (2003) Inactivation of the small GTPase Rac1 protects the liver from ischemia/reperfusion injury in the rat. Surgery 134: 480–491. 14555937 10.1067/s0039-6060(03)00256-3

[14] Yao HY, Chen L, Xu C, Wang J, Chen J, et al (2011) Inhibition of Rac activity alleviates lipopolysaccharide-induced acute pulmonary injury in mice. Biochim Biophys Acta 1810: 666–674. 21511011 10.1016/j.bbagen.2011.03.020

[15] Binker MG, Binker-Cosen AA, Gaisano HY, Cosen-Binker LI (2008) Inhibition of Rac1 decreases the severity of pancreatitis and pancreatitis-associated lung injury in mice. Exp Physiol 93: 1091–1103. 18567599 10.1113/expphysiol.2008.043141

[16] Soehnlein O, Oehmcke S, Ma X, Rothfuchs AG, Frithiof R, et al (2008) Neutrophil degranulation mediates severe lung damage triggered by streptococcal M1 protein. Eur Respir J 32: 405–412. 18321926 10.1183/09031936.00173207

[17] Kamochi M, Kamochi F, Kim YB, Sawh S, Sanders JM, et al (1999) P-selectin and ICAM-1 mediate endotoxin-induced neutrophil recruitment and injury to the lung and liver. Am J Physiol 277: L310–319. 10444525 10.1152/ajplung.1999.277.2.L310

[18] Asaduzzaman M, Zhang S, Lavasani S, Wang Y, Thorlacius H (2008) LFA-1 and MAC-1 mediate pulmonary recruitment of neutrophils and tissue damage in abdominal sepsis. Shock 30: 254–259. 18197144 10.1097/shk.0b013e318162c567

[19] Asaduzzaman M, Rahman M, Jeppsson B, Thorlacius H (2009) P-selectin glycoprotein-ligand-1 regulates pulmonary recruitment of neutrophils in a platelet-independent manner in abdominal sepsis. Br J Pharmacol 156: 307–315. 19154425 10.1111/j.1476-5381.2008.00021.x PMC2697831

[20] Zhang S, Rahman M, Herwald H, Thorlacius H (2011) Streptococcal M1 protein-induced lung injury is independent of platelets in mice. Shock 35: 86–91. 20577151 10.1097/SHK.0b013e3181ea4476

[21] Collin M, Olsen A (2000) Generation of a mature streptococcal cysteine proteinase is dependent on cell wall-anchored M1 protein. Mol Microbiol 36: 1306–1318. 10931281 10.1046/j.1365-2958.2000.01942.x

[22] Hasan Z, Rahman M, Palani K, Syk I, Jeppsson B, et al (2013) Geranylgeranyl transferase regulates CXC chemokine formation in alveolar macrophages and neutrophil recruitment in septic lung injury. Am J Physiol Lung Cell Mol Physiol 304: L221–229. 23241528 10.1152/ajplung.00199.2012

[23] Zhang S, Rahman M, Jeppsson B, Herwald H, Thorlacius H (2013) Geranylgeranyl transferase regulates streptococcal m1 protein-induced CXC chemokine formation and neutrophil recruitment in the lung. Shock 39: 293–298. 23364431 10.1097/SHK.0b013e3182844523

[24] Zhang S, Rahman M, Jeppsson B, Herwald H, Thorlacius H (2012) Streptococcal m1 protein triggers farnesyltransferase-dependent formation of CXC chemokines in alveolar macrophages and neutrophil infiltration of the lungs. Infect Immun 80: 3952–3959. 22949548 10.1128/IAI.00696-12 PMC3486054

[25] Schramm R, Thorlacius H (2004) Neutrophil recruitment in mast cell-dependent inflammation: inhibitory mechanisms of glucocorticoids. Inflamm Res 53: 644–652. 15654511 10.1007/s00011-004-1307-8

[26] Zhang S, Rahman M, Wang Y, Herwald H, Jeppsson B, et al (2012) p38 Mitogen-activated protein kinase signaling regulates streptococcal M1 protein-induced neutrophil activation and lung injury. J Leukoc Biol 91: 137–145. 21971519 10.1189/jlb.0511268

